# Departments Which Do Not Always Pay

**Published:** 1912-04-20

**Authors:** 


### INSTITUTIONAL HOUSEKEEPING.

### Departments which do not always Pay.

### I.—THE LAUNDRY.

The tendency in the modern hospital is more and more to be self-sufficing. One department after another is added to the activities undertaken, and it becomes increasingly important to make certain that each in turn is self-supporting and repays the labour bestowed on it. Undoubtedly the responsibilities of the superintendent, whether matron or secretary, are heavily added to by these outlying provinces, which demand expert knowledge of many matters outside ordinary hospital routine, and it is not certain that their time, with all the other calls on their attention, will admit of their managing these outside concerns successfully. Modern methods of finance, under the stimulus of the Uniform System of Accounts, enable managers to ascertain at a glance whether it is worth while, from a monetary point of view, to place this additional burden on the superintendent, whether the hospital gains or loses by undertaking its own business. There can be little doubt that in numerous instances the account balances out against the hospital, and that such enterprises are sometimes a complete failure.

It is essential to remember, however, that a hospital department may pay without being an economy in money. It is instructive to glance at the cost of washing in London hospitals as revealed in the report of the King's Fund. It will be seen that some of the hospitals which run their own laundries pay considerably more per dozen articles than others which contract for their washing outside. The Middlesex Hospital, for instance, paid at the rate of 10.09 pence per dozen for washing done in their fine laundry at Hendon in 1910, as compared with the rate of 7.74 pence per dozen paid by St. Mary's, Paddington. When the accounts are examined it is seen that the cost is swelled by interest on capital expended on buildings and machinery, by heavy items reckoned off for depreciation of plant, and by cost of carriage to and from the hospital. It is impossible for hospitals to compete with the ordinary commercial laundry unless they are able to construct the necessary buildings in the hospital grounds, as has been done at Guy's and at the London Hospital. But it does not follow that the Middlesex Hospital loses by the increased cost of the washing since the new laundry was built. There are the strongest possible reasons why a hospital containing cancer and maternity departments, in addition to general medical and surgical wards, should incur an increased outlay in order to ensure that the laundry is conducted on the best possible lines. The construction of the laundry was one of the achievements of that far-sighted administrator, the late Mr. Edward Fardon, to whom the Middlesex Hospital owes so much, and it was built in full knowledge of the fact that it involved extra expenditure. At the London Hospital laundry, however, situated in the hospital grounds, the cost has been reduced to the astonishing figure of 6.67 pence per dozen articles, which is considerably less than that paid for washing off the premises in any large general hospital in London.

It is quite possible, however, for money to be wasted on running a laundry. When comparison with other institutions reveals that more is being spent on this department than in other laundries of its own class, it becomes necessary to inquire into the causes of the leakage. At the Royal Free Hospital, some years ago, the cost of the laundry was increased to an alarming extent by a blunder in the size of the boiler, not easy to rectify. When superintendence is slack, much may be wasted on materials. When labour is scarce it may be more satisfactory to contract than to meet the perpetual difficulties involved in training poor hands. And, unless there be energy and capacity to spare at headquarters for the general supervision of the laundry in all its departments, it can never really pay the hospital to undertake this branch of work. For a hospital laundry not under direct inspection and direction from the hospital itself is on the same footing as a commercial laundry, save that it lacks the stimulus which is afforded by the ever-pressing necessity for earning a dividend, and hence for keeping things to a certain extent up to a good standard of efficiency. It is far better to have the laundry well managed under the contract system than to muddle on expensively with a badly conducted private enterprise which no one has time or knowledge to direct.

### HOUSEKEEPING QUERIES.

*Under this heading questions are dealt with relating to cookery and household recipes, the duties of the domestic staff, estimates of quantities required for given numbers, and the cost of diet for patients and staff. Arrangements have been made to deal with samples submitted for examination and report on them.*

### Frozen Meat.

"Matron": Very glad to know that you "follow the housekeeping articles with great interest, especially the recent ones on prices, which are certainly astonishing." We are very pleased to have your article, which will be paid for at our usual rates when published. A further communication will be welcomed.

### To Keep Milk Sweet.

"Temporary Matron": Our committee are not satisfied with the way in which we keep our milk. We have no refrigerators in the ward kitchens, and the milk is not sent up to the wards in cans, but is kept in the dairy. Do you advise our keeping it in enamel vessels or in tin ones, and should there be lids or not? There is a good deal of coming and going in our dairy, as we have to keep other things there besides milk.

